# Molecular analysis of the apoptotic effects of BPA in acute myeloid leukemia cells

**DOI:** 10.1186/1479-5876-7-48

**Published:** 2009-06-18

**Authors:** Paola Bontempo, Luigi Mita, Antonella Doto, Marco Miceli, Angela Nebbioso, Ilaria Lepore, GianLuigi Franci, Roberta Menafra, Vincenzo Carafa, Mariarosaria Conte, Floriana De Bellis, Fabio Manzo, Vincenzo Di Cerbo, Rosaria Benedetti, Loredana D'Amato, Maria Marino, Alessandro Bolli, Giovanna Del Pozzo, Nadia Diano, Marianna Portaccio, Gustavo D Mita, Maria Teresa Vietri, Michele Cioffi, Ernesto Nola, Carmela Dell'Aversana, Vincenzo Sica, Anna Maria Molinari, Lucia Altucci

**Affiliations:** 1 Dipartimento di Patologia generale, Seconda Università di Napoli, Via L. De Crecchio 7 Napoli, Italy; 2 Istituto Nazionale di Biostruttura e dei Biosistemi, Viale Medaglie d'Oro,305, 00100 Roma, Italy; 3 Dipartimento di Medicina sperimentale, Seconda Università di Napoli, Via De Crecchio, Napoli, Italy; 4 Dipartimento di Fisica, Università di Napoli 'Federico II', Napoli, Italy; 5 Dipartimento di Biologia, Università Roma Tre, Viale Guglielmo Marconi 446, 00146 Roma, Italy; 6 Istituto di Genetica e Biofisica del CNR, Via P. Castellino 111, 80100 Napoli, Italy

## Abstract

**Background:**

BPA (bisphenol A or 2,2-bis(4-hydroxy-phenol)propane) is present in the manufacture of polycarbonate plastic and epoxy resins, which can be used in impact-resistant safety equipment and baby bottles, as protective coatings inside metal food containers, and as composites and sealants in dentistry. Recently, attention has focused on the estrogen-like and carcinogenic adverse effects of BPA. Thus, it is necessary to investigate the cytotoxicity and apoptosis-inducing activity of this compound.

**Methods:**

Cell cycle, apoptosis and differentiation analyses; western blots.

**Results:**

BPA is able to induce cell cycle arrest and apoptosis in three different acute myeloid leukemias. Although some granulocytic differentiation concomitantly occurred in NB4 cells upon BPA treatment, the major action was the induction of apoptosis. BPA mediated apoptosis was caspase dependent and occurred by activation of extrinsic and intrinsic cell death pathways modulating both FAS and TRAIL and by inducing BAD phosphorylation in NB4 cells. Finally, also non genomic actions such as the early decrease of both ERK and AKT phosphorylation were induced by BPA thus indicating that a complex intersection of regulations occur for the apoptotic action of BPA.

**Conclusion:**

BPA is able to induce apoptosis in leukemia cells via caspase activation and involvement of both intrinsic and extrinsic pathways of apoptosis.

## Background

The Endocrine Disrupting Compounds are defined as "*exogenous substances that cause adverse health effects in an intact organism, or its progeny, secondary to changes in endocrine function*" (EEC, 1996). Their effects on humans, wildlife and the environment have been subject of high attention by the scientific community, since concerns were first raised about them by Colborn [1]. Recently, the potential of certain pesticides to act as EDCs has been confirmed. These include organometallic compounds, and many other organochlorine compounds that are also toxic and persistent [2,3], and many have been banned as a result [2]. Other pesticides such as organophosphates, carbamates, triazines and pyrethroids that are less persistent and less toxic than the organochlorines, were used to replace them, but many are now confirmed or suspected EDCs [4]. Conventional toxicological testing of pesticides can miss the potential of a substance to disrupt the endocrine system, especially at the low concentrations likely to be found in the environment. It is generally assumed that chemical substances will show a simple monotonic dose- response curve, but some ED pesticides have j-type dose- response curves [5], whereby the toxic effects decrease as the dose decreases, until at very low doses (often as low as parts per billion or even trillion) their effects increase [5]. Of the more than 2,000 high-production volume chemicals that are manufactured in or imported many are widely used in consumer products. Among the many chemicals is bisphenol A [BPA; 2,2-bis(4-hydroxyphenyl)propane]. BPA is used in the manufacture of polycarbonate plastic and epoxy resins, which can be used in impact-resistant safety equipment and baby bottles, as protective coatings inside metal food containers, and as composites and sealants in dentistry. Exposure to BPA is thought to result primarily from ingestion of food containing BPA [6,7]. At high doses, BPA demonstrates estrogen-like effects on uterine and prostate organ weights in experimental animals. At doses below the putative lowest observed adverse effect level, exposure to BPA has resulted in decreased sperm production, increased prostate gland volume, altered development and tissue organization of the mammary gland, altered vaginal morphology and estrous cycles, disruption of sexual differentiation in the brain, and accelerated growth and puberty [8-16]. BPA is of concern to environmental public health because of the high potential for exposure of humans to these phenols and their demonstrated animal toxicity. Recently, attention has focused on the carcinogenic adverse effects of BPA. Thus, it is important to investigate the cytotoxicity and apoptosis-inducing activity of these compounds [17,18]. In the present manuscript, we decided to investigate the effects of different doses of BPA on acute myeloid leukemia models to understand the mechanism(s) of BPA action in systems not directly related to the endocrine system. We show indeed that BPA is able to induce apoptosis in leukemia cells by activation of the initiator caspases 8, 9 and the effector caspases 3-7. Moreover we show that many genomic and non-genomic players are influenced by the action of BPA and contribute to its adverse effects.

## Methods

### Cell lines

All cell lines have been obtained from ATCC and routinely cultured. NB4, U937, k562, and cells HL60, were grown at 37°C in air and 5% CO2 in RPMI 1640 medium (GIBCO), supplemented with 10% heat-inactivated foetal bovine serum (FBS), 1% l-glutamine, 1% ampicillin/streptomycin and 0, 1% gentamicin. BPA (SIGMA) was resuspended in ethanol and at the final concentration of 1 μM. All *trans *retinoic acid (SIGMA) (RA) was resuspended in ethanol and at the final concentration of 1 μM. To understand the potential role of BPA leukemia cell lines were treated with different concentrations of BPA (10, 30, 60, 100 μM) for different times.

### Cell cycle analysis

2.5 × 10^5 ^cells were collected and resuspended in 500 μl of a hypotonic buffer (0.1% Triton X-100, 0.1% sodium citrate, 50 μg/ml propidium iodide (PI), RNAse A). Cells were incubated in the dark for 30 min. Samples were acquired on a FACS Calibur flow cytometer using the Cell Quest software (Becton Dickinson) and analysed with standard procedures using the Cell Quest software (Becton Dickinson) and the ModFit LT version 3 Software (Verity) as previously reported [19]. All the experiments were performed in triplicate.

### FACS analysis of apoptosis

Apoptosis was measured with Annexin V/PI double staining detection (Roche and Sigma-Aldrich, respectively) as recommended by the suppliers; samples were analysed by FACS with Cell Quest technology (Becton Dickinson) as previously reported [20,21]. We measured as apoptotic fraction the Annexin V positive, PI negative cells. As second assays the caspase 8, 9 and 7, 3 detection (B-Bridge) was performed as recommended by suppliers and quantified by FACS (Becton Dickinson). NB4 cells were treated for 48 h with 10-60-100 μM BPA.

### Granulocytic differentiation assay

Granulocytic differentiation was carried out as previously described [22]. Briefly, NB4 cells, treated for 48 h with 10-30-60-100 μM BPA, ATRA 1 μM or with ATRA 1 μM and BPA at the indicate prima concentrations, were harvested and resuspended in 10 μl phycoerythrine-conjugated CD11c (CD11c-PE) (Pharmingen). Control samples were incubated with 10 μl PE or FITC conjugated mouse IgG1, incubated for 30 min at 4°C in the dark, washed in PBS and resuspended in 500 μl PBS containing PI (0.25 μg/ml). Samples were analysed by FACS with Cell Quest technology (Becton Dickinson). PI positive cells have been excluded from the analysis.

### Western blot analyses

40 micrograms of total protein extracts were separated on a 15% polyacrylamide gel and blotted as previously described [23]. Western blots were shown for p21 (Transduction Laboratories, dilution 1:500), p27 c-19 (Santa Cruz sc-528 rabbit, dilution 1:500), p16 (Santa Cruz sc-468 rabbit, dilution 1:500). For determination of Rb, pRb, p53, ERalpha and cyclin D 35 μg of total protein extracts were separated on a polyacrylamide gel and blotted. Antibodies were: cyclin D (Zymed), pRb, p53, RB and ERalpha (Santa Cruz). Total ERKs (Santa Cruz) were used to normalise for equal loading. For quantification of TRAIL protein, 100 μg of total protein extracts were separated on a 10% polyacrylamide gel and blotted. Western blots were shown for TRAIL (Abcam Ab 16963-1). For determination of FAS, FLIP-L and FLIP-S, BAD, pBAD and BCL2, 35 μg of total protein extracts were separated on a 12% polyacrylamide gel and blotted. Antibodies used were: FAS (ProSci xw-7192, dilution 1:500), Flip (Alexis 804-429-C100, dilution 1:500), BAD (Cell signalling #9292, dilution 1:500), pBAD (p-Bad ser 136, #9295 cell signalling, dilution 1:500), Bcl2 (Bcl2 (Ab-1) Oncogene Science, dilution 1:500). Total ERKs were used to normalise for equal loading.

For determination of ERK2, pERK, Akt and pAkt, 35 μg of total protein extracts were separated on a 12% polyacrylamide gel and blotted. Antibodies used were: ERK2 (Santa Cruz sc-154, dilution 1:500), pERK (Santa Cruz sc-7383, dilution 1:200), pAkt (Cell signalling cod 9271, dilution 1:1000) and Akt (Cell signalling Akt cod 9272, dilution 1:1000). For quantification of histone H3 acetylation, 40 μg of total protein extracts were separated on a 15% polyacrylamide gel and blotted. Antibodies used were: acetylated histone H3 (Upstate cat. 06-599, dilution 1:500). Total ERKs were used to normalise for equal loading.

## Results

### BPA induces dose dependent apoptosis in acute myeloid leukemia cells

**Figure 1:**
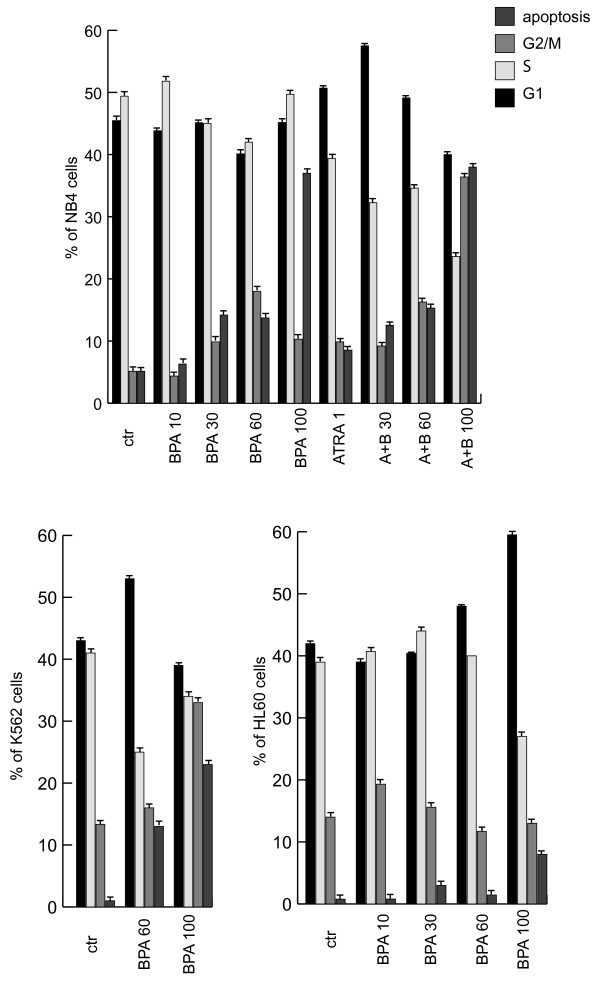
**BPA induces dose dependent apoptosis and cell cycle block in acute myeloid leukemia cells**. (A) Cell cycle and apoptosis in NB4 cells after treatment with 10,30,60 and 100 μM BPA, ATRA (all-*trans*-retinoic acid) 1 μM and the combination of ATRA 1 μM and BPA, at the indicated concentrations for 48 hrs. (B) Cell cycle analysis and apoptosis in K562 cells after 48 hrs of treatment with 60 and 100 μM BPA. (C) Cell cycle analysis and apoptosis in HL60 cells after treatment with 10, 30, 60 and 100 μM BPA for 48 hrs.

To understand the potential role of BPA in biological systems of leukemias we tested the action of BPA in three different acute myeloid leukemia models such as NB4, HL60 and K562 cells. As it is shown in Fig. 1, different concentrations of BPA are able to induce an increase of the sub-G1 peack in all the cell lines tested, HL60 being the most resistant one. In NB4 cells, a model from promyelocytic leukemia containing the fusion protein PML-RARα and sensitive to retinoids, the highest concentration of BPA used induces around 38% of apoptosis after 48 hrs. This apoptosis is not synergistically modulated by the double treatment with 1 μM Retinoic Acid (RA) as shown in Fig. 1A. Differently, cell cycle arrest seems to be affected by the double treatment, showing an increase of the G1 peack at low dose BPA (30 μM) and an increase of the G2-M fraction of cells at the highest concentration of BPA (100 μM). Differently, in the K562 cells, a model of AML derived from a CML containing the Philadelphia chromosome, the treatment with BPA showed an increase of cell death proportional to the dose increase of BPA, together with a G1 peack at the lower dose and a G2-M increase at the higher dose (Fig. 1B). Finally, HL60 cells showed an increase of apoptosis at the higher dose of BPA (100 μM) in agreement with what reported previously [17]. This increase is directly proportional with the enrichment in G1 phase of HL60 cells upon treatment with increasing doses of BPA (Fig. 1C).

### BPA induces dose dependent differentiation in NB4 cells

**Figure 2:**
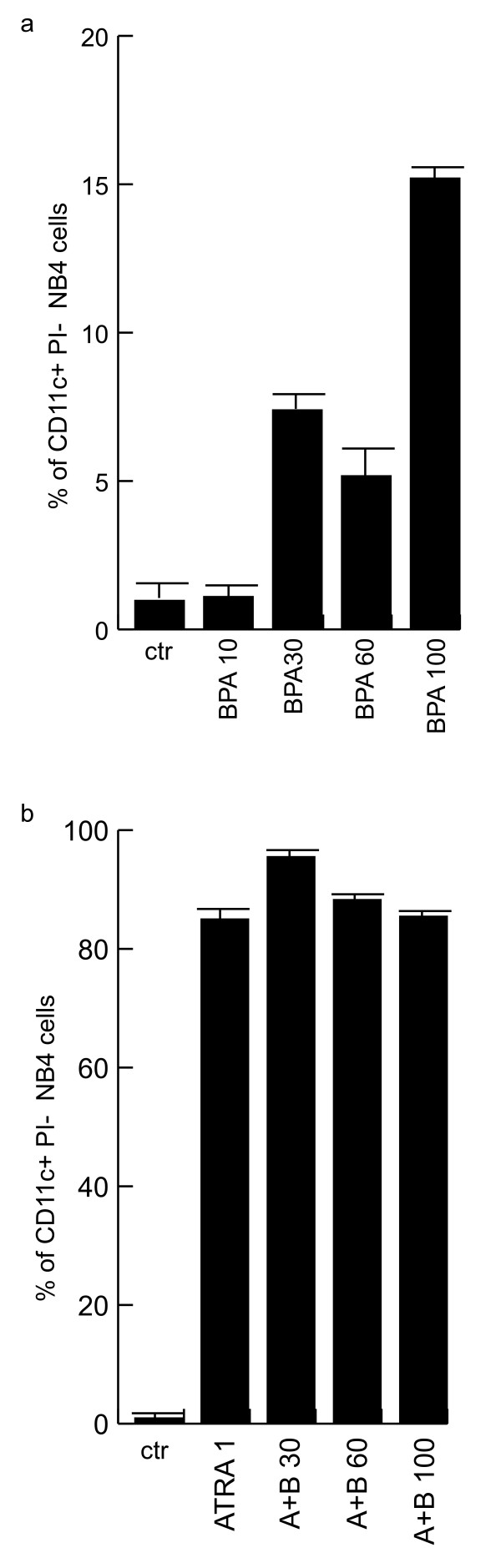
**BPA induces dose dependent differentiation in NB4 cells**. (A) CD11c expression levels measured by FACS after 48 h of treatment with 10,30,60 and 100 μM BPA. (B) CD11c expression levels after treatment with ATRA 1 μM or with the combination of ATRA 1 μM and BPA at the indicated concentrations for 48 hrs. Note that PI positive cells have been excluded from the analysis.

That BPA was able to induce apoptosis and to influence the cell cycle of NB4 cells, prompted us to check its effects on granulocytic differentiation of these cells. As shown in Fig. 2A by FACS analyses, BPA is able to differentiate NB4 cells versus granulocytes in a dose dependent manner. However, the effect was weak if compared with the one of RA at the same time in the NB4 cells (Fig. 2B), thus showing that BPA preferentially activates apoptotic actions in respect to differentiative effects in these cells.

### BPA induces apoptosis via caspase activation in NB4 cells

**Figure 3:**
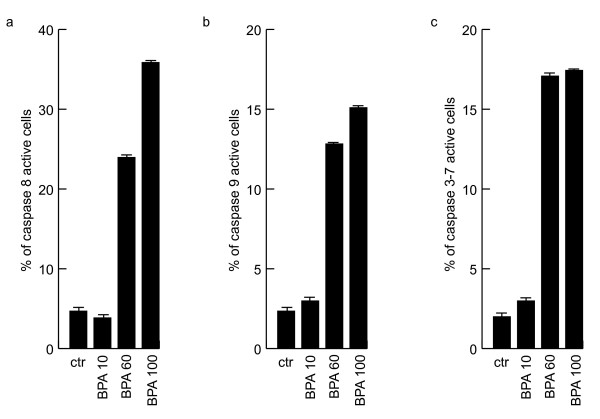
**BPA induces apoptosis via caspase activation in NB4 cells**. Caspase 8, 9 and 3–7 assays have been carried out by FACS analysis in NB4 cells after 48 h of incubation with the indicated concentrations of BPA.

To better identify which apoptotic pathway is activated by BPA, we tested by FACS analyses the initiator and effector caspases activation in NB4 cells after 48 h treatment with BPA. As it is shown in Fig. 3, both caspase 8 (Fig. 3A) and 9 (Fig. 3B) are cleaved and active upon BPA treatment. Note that caspase 8 resulted more active, suggesting a prior activity of BPA on the extrinsic pathway of apoptosis at least as time scale. As expected, caspase 3–7, which are downstream of caspase 8 and 9, resulted activated by medium (60) and high doses (100) of BPA.

### BPA induces modulation of cell cycle regulators and apoptotic players in NB4 cells

**Figure 4:**
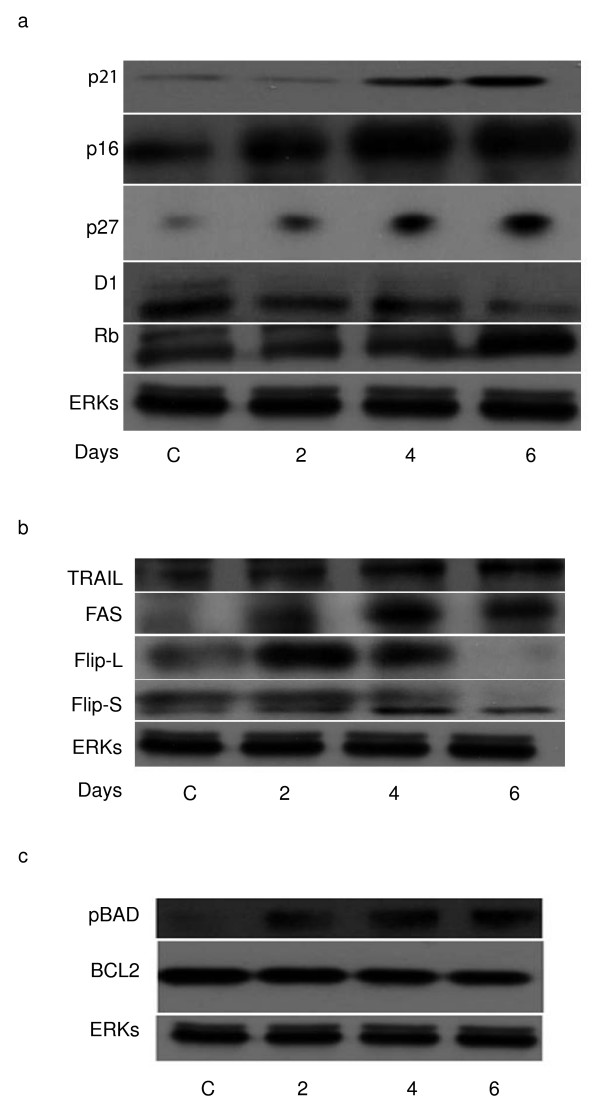
**BPA induces modulation of cell cycle regulators and apoptotic players in NB4 cells**. (A) Western blot analysis showing p21, p27, p16, cyclin D1 and RB expression levels in NB4 cells treated with 60 μM BPA for 2, 4 and 6 days. (B) Western blot analysis showing TRAIL, FAS, Flip-L and Flip-S expression levels in NB4 cells treated with 60 μM BPA for the indicated days. (C) Western blot analysis showing BCL2 and pBAD expression levels after treated with 60 μM BPA for the indicated days. Total ERKs expression levels account for equal loading.

That BPA influenced both cell cycle progression and apoptosis of acute myeloid leukemias has been clarified by these results. To understand which molecular events underlie to these effects, we have tested its action on known cell cycle regulators in NB4 cells in a time dependent manner. As shown in Fig 4A, p21, p27 and p16 together with RB are up-regulated by BPA at the 60 μM dose, whereas cyclin D1 which is known to modulate proliferation gets decreased. This scenario is reminiscent of a cell cycle block regulated at the molecular level. At the same time, checking for apoptotic key players we found that both FAS and TRAIL are up-regulated already at day 2 of treatment, while Flip-L is transiently up-regulated and then down-regulated, whereas Flip-S is down regulated (Fig. 4B). At the mitochondria cell death level, we could not find modulation of BCL2, but we could see increased phosphorylation of BAD (Fig. 4C) thus confirming that both pathways (extrinsic and intrinsic) gets activated by BPA in NB4 cells.

### BPA induces modulation of ERK, AKT and Rb phosphorylation and increase of histone acetylation in NB4 cells

**Figure 5:**
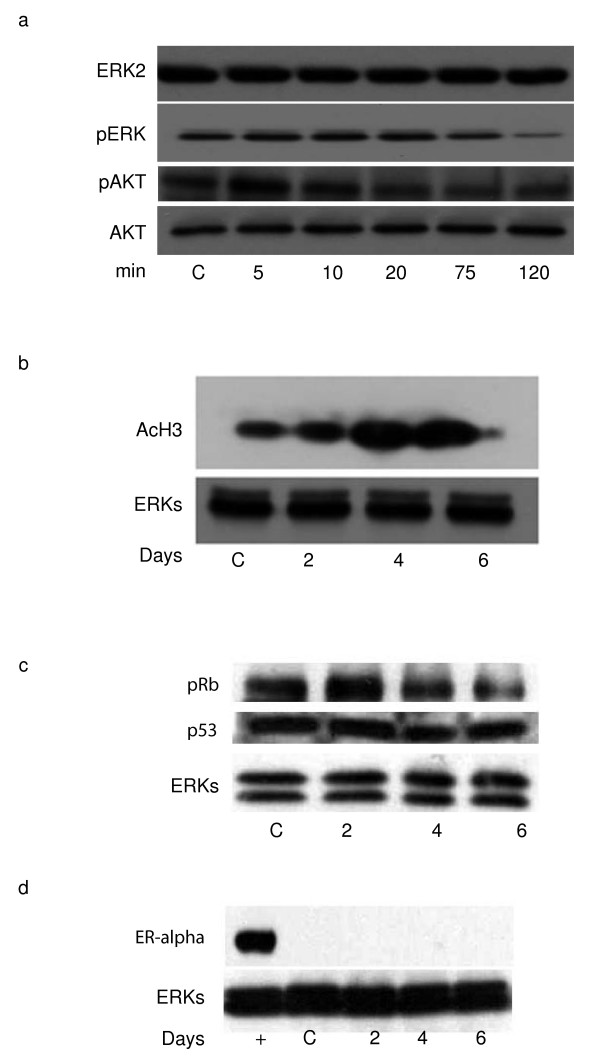
**BPA induces modulation of ERK and AKT phosphorylation and increase of histone acetylation in NB4 cells**. (A) Western blot analysis showing ERK and AKT phosphorylation in NB4 cells treated with 60 μM BPA at the times indicated times; (B) Western blot analysis of the acetylation levels of Histone H3 in NB4 cells treated for 2, 4 and 6 days with 60 μM BPA. ERKs expression levels account for equal loading); (C) Western blot analysis of the phosphorylation levels of Rb and p53 expression in NB4 cells treated for 2, 4 and 6 days with 60 μM BPA. ERKs expression levels account for equal loading); (D) Western blot analysis of the expression levels of ER alpha in NB4 cells treated for 2, 4 and 6 days with 60 μM BPA. As positive control for the ER alpha detection (indicated as +) 25 μg of MCF7 protein extracts have been used. ERKs expression levels account for equal loading.

To better focus the activity of BPA in acute myeloid leukemia models, we decided to check whether BPA can also modulate non genomic actions. As shown in Fig. 5, BPA induce a decrease of ERK, Rb and AKT phosphorylation thus indicating that anti-proliferative actions occur by induction of non genomic pathways by 60 μM of BPA in NB4 cells. Note that p53 expression levels stayed unchanged (Fig. 5c). In agreement with these findings, histone H3 acetylation is increased upon BPA treatment suggesting an effect (direct or indirect) on chromatin accessibility of BPA (Fig. 5B).

## Discussion

The Endocrine Disrupting Compounds have been subject of high attention by the scientific community, since concerns have been raised about their actions and potential toxicities. Among the many chemicals, BPA is used in the assemble of polycarbonate plastic and epoxy resins, used in impact-resistant safety equipment and baby bottles, as protective coatings inside metal food containers, and as composite and sealant in dentistry. Exposure to BPA is thought to result primarily from ingestion of food containing BPA [6,7]. BPA is of concern to environmental public health because of its toxicity. At high doses, BPA demonstrates estrogen-like effects in experimental animals, but effects independent from its endocrine modulating function have been poorly investigated. Thus, it is central to investigate the cyto-toxicity and apoptosis-inducing activities of BPA at the molecular level. The fact that BPA is able to induce effects on cell cycle and apoptosis in AML models indicates that BPA actions can go beyond the endocrine interference. This is also demonstrated by the fact that NB4 cells do not display detectable levels of ER alpha. Thus suggesting that effects of BPA in this cells are largely ER independent (Fig. 5d). This notion is a key point considering that BPA is industrially used and that its effects can cumulate. Although the properties seen on granulocytic differentiation are minor when compared to those of RA, the fact that BPA is used in equipments and baby bottles makes also these weak effects of significance. Even more interesting is the induction of cell death which is clearly specifically regulated at the molecular level. Indeed, the fact that three different cell lines respond with apoptosis to BPA treatment and that this effect seems to be dose dependent indicates that this is a general feature of BPA treatment and that this might be reproduced in many other cells. These evidences are exciting from several point of view: if from one side we might consider the induction of apoptosis as an interesting anti-cancer action, on the other side we have to keep in mind that these effects might also be elicited in normal cells in the different compartments of the human body and thus might contribute to the toxicity of BPA. The regulation of caspase-dependent pathways of apoptosis suggests a specific action on the extrinsic and intrinsic pathways of apoptosis which is confirmed by the clear induction of Fas and TRAIL and by Flip down regulation in NB4 cells. Even if our data would support a model in which the extrinsic pathway of apoptosis is more active, we do not exclude the importance of the mitochondria de-regulation of apoptosis which is indeed confirmed by caspase 9 activation and BAD phosphorylation. Considering that many clinical treatments target apoptosis at the present, our data suggest that the contact or the assumption of BPA might increase the effects of a on-going treatment in humans, apart, of course, having effects on its own. Finally, the fact that BPA decreases the activity of ERK and AKT well integrates with its anti-proliferative and apoptotic actions suggesting that the cross-talk of different molecular actions contribute to the cell cycle arrest and to the apoptosis in human biological systems. The hyperacetylating effect shown on histone H3 confirms the property of BPA to modulate the chromatin in a more accessible state thus corroborating the hypothesis that BPA contributes with a plethora of different effects to the induction of cell cycle arrest, weak differentiation and apoptosis in a specific and molecularly defined manner. If the hyperacetylation upon BPA treatment is a direct or indirect effect on chromatin, remains to be established. More characterized studies on BPA exposed population in healthy or unhealthy status will decipher in the future the real impact of these molecular actions.

## Conclusion

Our data strongly indicate that BPA has molecular activities that go much beyond its ED function. These actions have been well focused as cell cycle arrest and apoptosis and the molecular pathways involved have been identified. This knowledge clearly shows that BPA effects have to be considered independently of its ED action and might help in the understanding of the adverse effects caused in humans.

## Abbreviations

AKT: RAC-alpha serine/threonine-protein kinase; AML: Acute Myeloid Leukemia; ATRA: All Trans Retinoic Acid; BAD: Bcl2 Antagonist of cell Death, BCL2-associated death promoter; BCL2: B Cell Lynphoma 2; BPA: Bisphenol A or 2,2-bis(4-hydroxy-phenol)propane; CML: Chronic Myeloid Leukemia; EDC: Endocrine Disruptor Compounds; ED: Endocrine Disruptor; ERK: Extracellular Signal-Regulated Kinase; FAS: Apoptosis-mediating Surface Antigen, Tumor necrosis factor receptor superfamily member 6; FLIP: FLICE Inhibitor Protein; TRAIL: TNF Related Apoptosis Inducing Ligand; RA: Retinoic Acid; RB: Retinoblastoma.

## Competing interests

The authors declare that they have no competing interests.

## Authors' contributions

PB, LM, AD, MM, AN, IL, GF, RM, VC, MC, FDB, FM, CDA, VDC, MM, AB, GDP, ND, M, LD, MTV, MC, RB, EN, VS, GDM and AM contributing in performing the experiments shown and in the conceptual understanding of the results. PB and LA critically discussed the experimental data and wrote the manuscript.
